# Impairment-targeted exercises for older adults with knee pain: protocol for a proof-of-principle study

**DOI:** 10.1186/1471-2474-12-2

**Published:** 2011-01-07

**Authors:** Laurence RJ Wood, George M Peat, Ricky Mullis, Elaine Thomas, Nadine E Foster

**Affiliations:** 1 Arthritis Research UK Primary Care Centre, Keele University, Keele, Staffordshire, ST7 8AE, UK; 2 General Practice and Primary Care Research Unit, Institute of Public Health, University of Cambridge, IPH, Forvie Site, Robinson Way, Cambridge, Cambridgeshire, CB2 0SR, UK

## Abstract

**Background:**

Exercise therapy for knee pain and osteoarthritis remains a key element of conservative treatment, recommended in clinical guidelines. Yet systematic reviews point to only modest benefits from exercise interventions.

One reason for this might be that clinical trials tend to use a one-size-fits-all approach to exercise, effectively disregarding the details of their participants' clinical presentations. This uncontrolled before-after study (TargET-Knee-Pain) aims to test the principle that exercises targeted at the specific physical impairments of older adults with knee pain may be able to significantly improve those impairments. It is a first step towards testing the effectiveness of this more individually-tailored approach.

**Methods/Design:**

We aim to recruit 60 participants from an existing observational cohort of community-dwelling older adults with knee pain. Participants will all have at least one of the three physical impairments of weak quadriceps, a reduced range of knee flexion and poor standing balance. Each participant will be asked to undertake a programme of exercises, targeted at their particular combination and degree of impairment(s), over the course of twelve weeks. The exercises will be taught and progressed by an experienced physiotherapist, with reference to a "menu" of agreed exercises for each of the impairments, over the course of six fortnightly home visits, alternating with six fortnightly telephone calls. Primary outcome measures will be isometric quadriceps strength, knee flexion range of motion, timed single-leg standing balance and the "Four Balance Test Scale" at 12 weeks. Key secondary outcome measures will be self-reported levels of pain, stiffness and difficulties with day-to-day functional tasks (WOMAC). Outcome measures will be taken at three time-points (baseline, six weeks and twelve weeks) by a study nurse blinded to the exercise status of the participants.

**Discussion:**

This study (TargET-Knee-Pain) is the first step towards exploring whether an impairment-targeted approach to exercise prescription for older adults with knee pain may have sufficient efficacy to warrant further testing. If warranted, future randomised clinical trials may compare this approach with more traditional one-size-fits-all exercise approaches.

**Trial registration:**

Current Controlled Trials ISRCTN61638364.

## Background

Knee pain, associated with osteoarthritis (OA), is a common disabling problem [1]. Most patients are managed in primary care, where exercise is considered to be a core first-line treatment [2]. Clinical guidelines support the overall effectiveness of exercise in knee and hip OA, but highlight the lack of evidence around the practical aspects of exercise delivery, including which exercises work best for whom [3-7]. Likewise, clinical trials support exercise programmes supervised by physiotherapists, in terms of reduction in knee pain and improvement in function [8-10]. However, systematic reviews often show, at best, small to moderate beneficial effects of exercise [6,7,11-14]. One potential reason for this may be that many of these clinical trials have tended to adopt a one-size-fits-all approach, whereby patients receive similar exercise interventions, regardless of the nature of their impairments. Instead, the MOVE Consensus has recommended that exercise therapy for OA of the hip or knee should be tailored to the individual patient [3]. One approach to doing this is to target exercises at specific and potentially-reversible physical impairments that are common in knee OA and are known to be associated with pain and disability [15-21]. But although evidence-based recommendations on rehabilitation interventions recommend strengthening, stretching and functional exercises, such as standing balance, for knee OA [22], there are currently no published trials that have specifically used an impairment-targeted exercise approach with this population.

In this paper, we present the protocol for a proof-of-principle study (TargET-Knee-Pain) to explore whether, and to what degree, an impairment-targeted approach to home-based exercise prescription can improve quadriceps strength, range of movement at the knee, and balance in older adults with knee pain and OA. This is the first stage towards determining the effectiveness of such impairment-targeted treatment approaches. As a secondary aim, this study will endeavour to determine to what degree any improvements in these factors may be reflected in corresponding improvements in self-reported knee pain, stiffness, and functional limitation.

## Methods/Design

### Design

Single-centre, uncontrolled before-after study.

### Setting

General population; based in participants' own homes.

### Sample

The sampling frame for this study will be existing participants in an established observational cohort study of knee pain and osteoarthritis, known as The Clinical Assessment Study of the Knee - CAS(K) [23,24]. CAS(K) participants were originally recruited from the registers of three general practices in North Staffordshire between 2002 and 2003. All were aged 50 years and older and reported knee pain within the previous 12 months. Participants have been followed up at 18-month intervals. We aim to recruit 60 participants to our study from a conservatively estimated population of 314 due to attend for repeat follow-up six years after their baseline assessments. Power calculations, based on observed effect sizes in previous trials of exercises for patients with OA of the knee, suggest that a sample size of 60 individuals would be capable of detecting an 8° improvement in the degree of knee flexion or an 8 Kg improvement in quadriceps strength with approximately 86% power, given a Type 1 error rate of 5% [25,26].

### Eligibility criteria

Potential participants in our study will be attendees at the CAS(K) 6-year-follow-up research assessment clinics, who are men and women, all aged 56+ years with a history of knee pain. To be included in this study, they will have measurements for at least one of the target impairments (quadriceps strength, degree of knee flexion and single-leg standing balance) that fall below the lowest quartile of age- and gender-stratified values recorded at the baseline assessments between 2002 and 2003 (Table 1). In order to be considered for inclusion in the study, individuals must be willing and able to commit to a programme of exercises for a 12-week period.

**Table 1:** Age and gender thresholds for study inclusion

	Male	Female
Range of knee joint flexion		
55-64 years	< 128°	< 127°
65-74 years	< 125°	< 122°
75+ years	< 120°	< 117°
		
Isometric quadriceps strength (kilograms force)		
55-64 years	< 18.1	< 11.3
65-74 years	< 17.2	< 9.4
75+ years	< 13.9	< 9.0
		
Single-leg standing balance (seconds)		
55-64 years	< 8	< 5
65-74 years	< 3	< 3
75+ years	< 2	< 2

The exclusion criteria are: total knee replacement of either knee joint; an existing diagnosis of inflammatory arthropathy; lower limb weakness from neurological conditions; receiving medication that adversely affects standing balance; open wounds on the anterior aspect of either distal shin; a self-report of unstable angina or uncontrolled hypertension/hypotension; an inner ear problem that compromises standing balance; no mobile or home telephone; unavailability for fortnightly home visits or telephone contact for the whole of a given working week of their potential involvement in the study; an inability to transfer independently from lying to sitting or from sitting to standing; currently receiving physiotherapy for their knee problem.

### Recruitment

Potential participants will be identified when they attend the CAS(K) 6-year follow-up research assessment clinics at a local community hospital. Their measurements for the three target impairments will be compared to the thresholds in Table 1. If a CAS(K) participant has a measurement for one or more of the target impairments that falls below one of the thresholds, he/she will be approached by the study nurse at the end of the research assessment clinic. The study nurse will explain the study in brief, provide the person with an information leaflet and seek permission to contact them by telephone to discuss their potential involvement in the study more fully.

**Figure 1:**
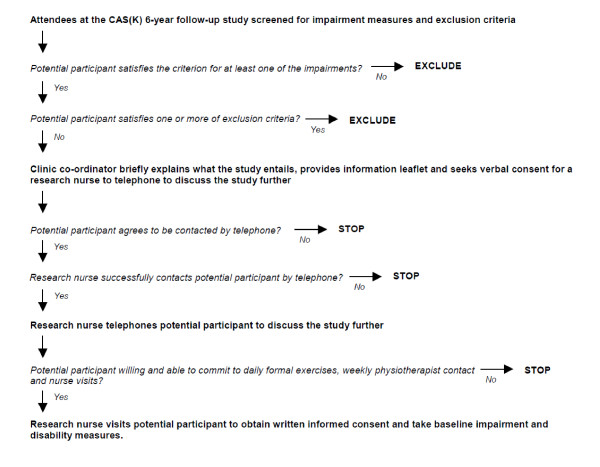
**Flowchart of recruitment process**.

If verbal consent to further telephone contact is given, the study nurse will contact potential participants, following a minimum 24 hour cooling-off period, in order to assess their willingness and suitability to participate. If an individual is willing and eligible to participate in the study, their agreement will be sought to arrange a time for the study nurse to visit them in their own home to undertake written consent and take baseline measurements. The recruitment process is summarised in the flowchart in Figure 1.

### Consent procedure

**Figure 2:**
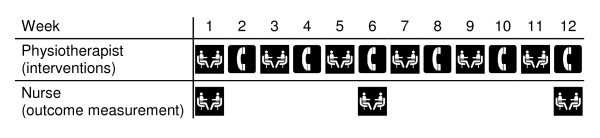
**Schematic diagram of physiotherapist and nurse home visits and telephone calls for the 12 weeks of each participant's involvement in the study**.

The study nurse will seek written informed consent at the initial home visit to each potential participant, explaining the study in detail and answering any questions. Consent will be sought for the following aspects of the study:

• Practising of daily home exercises, and keeping of a daily exercise diary.

• Study nurse visits in weeks 1, 6 and 12 of the study (Figure 2), involving measurement of quadriceps strengths, degree of knee flexion and standing balance, and completion of a self-report questionnaire, taking 10-20 minutes.

• Six fortnightly home visits from a physiotherapist (Figure 2), lasting approximately 45 minutes each, involving repeated measurement of the target impairment(s) and progression of the exercises, and alternating with fortnightly telephone calls (Figure 2).

### Interventions

In each case the study intervention will last for 12 weeks. Three exercise packages have been developed with reference to published literature (one for each of the three target impairments): one for quadriceps strengthening, one for knee flexion stretches, and one for balance retraining. These exercise packages systematically progress each of the exercises through photographically-illustrated stages. Details of the packages are available as Additional files 1, 2 and 3. Participants will be assigned to receive one or more of these exercise packages, based on whether their measurements for either leg for the corresponding target impairments fall below the thresholds in Table 1. The exercises will be selected and the level-of-difficulty tailored to the abilities of the individual participant on the basis of the assessment by a study physiotherapist during the first physiotherapist home visit. This assessment will consist of a standardised clinical history taking and physical assessment, together with measurement of the target impairment(s). Selection and tailoring of the exercises will take the following factors into account:

• The presence and degree of the three impairment(s).

• The safety of the participant.

• Participant ease of performance when practising the exercises under the supervision of the physiotherapist.

• The preferences, motivations and concerns of the individual participant.

The dosage and frequency of the exercises will be decided by negotiation with the individual participant. The physiotherapist will also employ the following elements of advice and reassurance when teaching the exercises to the participants:

• Explicit identification of activities of daily living that could be improved by an improvement in the identified impairment(s).

• Education regarding the importance of strong quadriceps and/or good flexion range of motion and/or good balance both to the health of the knee joints and to overall function.

• Advice and reassurance regarding the expected adverse effects of the exercises, such as temporary pain and stiffness, including advice about the benign nature of these side-effects and the use of ice, heat and simple analgesia to manage them.

• Reassurance that the individual can contact one of the study physiotherapists by telephone at any time regarding any queries or concerns.

Monitoring of participants' progress and appropriate progression of the exercise programme accordingly will be achieved through the fortnightly physiotherapist home visits, alternating with fortnightly telephone calls.

Adherence to the exercise programmes will be optimised in the following ways:

• By the physiotherapist cultivating a clinical partnership of shared decision-making with the participant that takes into account their individual preferences, motivations and concerns.

• By weekly physiotherapist contact with each of the participants, by either home visit or telephone conversation.

By an emphasis on positive reinforcement.

• By recording participants' progress with each of the relevant impairments at each home visit using pin-up wall-chart graphs.

• By encouraging participants to complete daily exercise diaries, which include details of which exercises were done how many times and how often, and any comments about the exercises and their performance.

In accordance with good clinical governance, details of the clinical history and findings from the physical examination of the participants will be recorded in writing in a standardised clinical notation booklet, together with participants' progress notes, so that the details of every physiotherapist contact with the participants are recorded.

### Outcome measures

All outcome measures will be administered by a study nurse entirely independently of the study physiotherapists. These measures will be taken at the first nurse home visit, following taking of written, informed consent, and again at the second and third nurse visits in weeks 6 and 12 of participants' involvement in the study. The outcome measures administered at all three time-points will be as follows:

#### Primary outcome measures - impairments

• Maximal isometric quadriceps strengths (continuous scale)

(measured in sitting at 90° knee flexion, using a Chatillon DFX-200 electronic dynamometer, stabilised against a wall, using a bespoke stabilisation rig (Additional file 4)).

• Degree of active end-range knee flexion (continuous scale).

(measured in supine on a portable examination couch, using a standard 10-inch universal perspex goniometer, with reference to the anatomical landmarks of the lateral malleolus of the ankle and the greater trochanter of the femur).

• The Four Balance Test Scale [27,28] (ordinal scale, range 0-5)

(tests the ability of the individual to balance in each of four postures for 10 seconds - feet together; semi-tandem stand; tandem stand and single leg stand).

• A modified version of Franchignoni et al's timed standing balance test [29,30] (continuous scale) (single-leg stance, hands on hips, up to a maximum of 30 seconds).

#### Secondary outcome measures - symptoms

• Self-report measures of pain, stiffness and physical function (WOMAC LK 3.1) [31].

• The persistence of knee pain symptoms (measured via one Likert response-type question) [32].

• The perceived 'bothersomeness' of the knee problem (measured via one Likert response-type question) [33].

In addition, the following secondary outcome measures, not gathered at baseline will be assessed at the 6 and 12 week nurse home visits:

• Global change in the knee problem (measured via Likert-response-type questions at 6 and 12 weeks) [34].

• Adherence to the exercise programme (measured via one Likert-response type question at 6 and 12 weeks, and via one free-text-response-type question at 12 weeks).

• Barriers to adherence to the exercise programmes, their acceptability and ways in which participants feel that they can be improved will be measured via two Likert-response-type questions and five free-text-response-type questions at 12-weeks only.

Additional information regarding adherence will be derived from the participant self-complete daily exercise diaries. Throughout the study, the study nurses taking the outcome measures will remain blinded to the impairment status of each of the participants.

### Data analysis

#### Primary analyses

We aim to test the hypothesis that simple home-based impairment-targeted exercises can improve those impairments in older adults with knee pain. To this end, all those participants receiving the exercise programme for a given impairment will have raw changes calculated for the measure of that impairment over the 12 weeks of the study. Mean differences in the change values (or their non-parametric equivalents, depending on the distribution of the change values) will then be calculated for each of the three impairment groups. Finally, in each instance, the mean differences in the change values for participants receiving an exercise programme targeted at a given impairment will be compared with those of participants not receiving an exercise programme targeted at that particular impairment, using the independent-samples t-test or its non-parametric equivalent.

#### Sensitivity analyses

To explore the potential contribution of regression to the mean, data on the three impairment measures collected for our participants at the baseline, 3-year and 6-year clinical assessment study clinics [23] will be compared to the corresponding measures taken at the initial TargET-Knee-Pain study nurse visit.

To explore the contribution of potential ceiling effects (i.e. how much improvement can be possible in the comparison groups, who will inevitably be starting with higher measures for the given target impairments), a definition of "lack of impairment" for each of the three impairments will be derived, based on age-gender stratified norms. The percentage of individuals who lack each of the target impairments, according to these definitions, will be determined at 12-week follow-up, and will be compared between those who were and those who were not impaired in that measure at recruitment.

To explore the effects that poor adherence with the exercise programmes may have on the results, efficacy subgroup analyses will be conducted, whereby the analyses will be repeated in the subgroup of individuals deemed to have been adherent with the exercise programmes and compared with the subset deemed not to have been adherent. For this purpose, inadequate adherence will be defined as follows:

• Not having done any of the exercises at all in the course of at least one rolling calendar week throughout the 12 week period of their involvement in the study (according to the daily exercise diaries).

• Not having done any of the exercises at all on a cumulative total of 12 days throughout the 12 week period of their involvement in the study (according to the daily exercise diaries).

• Participant self-report of not having done their exercises as often as they were advised to in either the 6 or 12 week questionnaires.

#### Secondary analyses

To investigate whether improvements in the target impairments are reflected in improvements in self-reported knee pain, stiffness and functional difficulties, mean changes (or their non-parametric equivalents, depending on the distribution of the change values) in the corresponding WOMAC subscale scores, between baseline and 12 weeks, will be calculated across all individuals, irrespective of which impairments they received exercises for. Associations between changes in the target impairments and changes in the WOMAC subscales will be examined by calculating Pearson's product-moment correlation coefficients (or their non-parametric equivalents, depending on the distribution of the change values).

To investigate whether improvements in self-report measures of pain, stiffness and physical function will be greater amongst those participants receiving exercises targeted at more than one impairment than amongst those participants receiving exercises targeted at only one impairment, mean differences in WOMAC change scores for the three subscales (or their non-parametric equivalents, depending on the distribution of the change values) will be calculated for both of these groups and compared across them.

To investigate the feasibility of this intervention and its acceptability to patients, responses to free-text and Likert-response-type questions will be analysed. This will include questions about barriers to adherence to the exercise programmes, and questions soliciting participants' views regarding participation in the study and the ways in which they believe that things could be improved. Analyses will include frequency counts of the various responses to Likert-type questions and a thematic analysis of free-text responses in the 12 week questionnaire.

#### Independent study monitoring

A study steering committee, with an independent chair and including lay-members of public, will be responsible for monitoring all aspects of the study at regular intervals, including any potential harms or adverse effects involving participants or research staff.

### Ethical approval

Ethical approval for this study to take place has been granted by the Black Country Research Ethics Committee (Ref. No. 08/H1202/179).

## Discussion

The TargET-Knee-Pain study will test the principle that a home-based exercise programme, specifically targeted at particular physical impairments of muscular weakness, decreased flexibility and poor balance, can positively influence those impairments in older adults with knee pain. This study is the first step towards exploring whether an impairment-targeted approach to exercise prescription for older adults with knee pain may have sufficient efficacy to warrant further testing. If warranted, future randomised clinical trials may compare this approach with more traditional one-size-fits-all exercise approaches.

## List of abbreviations used

TargET-Knee-Pain: Targeted Exercise Therapy for Knee Pain; WOMAC: Western Ontario & McMaster Universities Osteoarthritis Index; OA: osteoarthritis; CAS(K): The Clinical Assessment Study of the Knee; Kg: Kilograms.

## Competing interests

The authors declare that they have no competing interests.

## Authors' contributions

The idea for this study came from original work by LW. The study was jointly designed and funding secured by LW, GP, RM, ET and NF. All authors have been involved in drafting or revising the manuscript and have given final approval of the version to be published.

## Pre-publication history

The pre-publication history for this paper can be accessed here:

http://www.biomedcentral.com/1471-2474/12/2/prepub

## Supplementary Material

Additional file 1 **"Knee flexion stretch exercises"**. (Images of a model performing each of the exercises, together with a verbal description of how to perform each of the exercises). Click here for file

Additional file 2 **"Quadriceps strengthening exercises"**. (Images of a model performing each of the exercises, together with a verbal description of how to perform each of the exercises). Click here for file

Additional file 3 **"Balance retraining exercises"**. (Images of a model performing each of the exercises, together with a verbal description of how to perform each of the exercises). Click here for file

Additional file 4 **"Set-up for measurement of isometric quadriceps strengths"**. (Picture of the Chatillon DFX-200 electronic dynamometer with bespoke wall-stabilisation rig). Click here for file
