# Supplementary material for: Impairment-targeted exercises for older adults with knee pain: protocol for a proof-of-principle study
Source: BMC Musculoskelet Disord. 2011 Jan 7;12:2. doi: 10.1186/1471-2474-12-2 (PMC3025930; doi:10.1186/1471-2474-12-2)
Supplement: Additional file 1 — "Knee flexion stretch exercises". (Images of a model performing each of the exercises, together with a verbal description of how to perform each of the exercises). [file 1471-2474-12-2-S1.PDF]

STRETCH EXERCISES

	Picture	Description
A	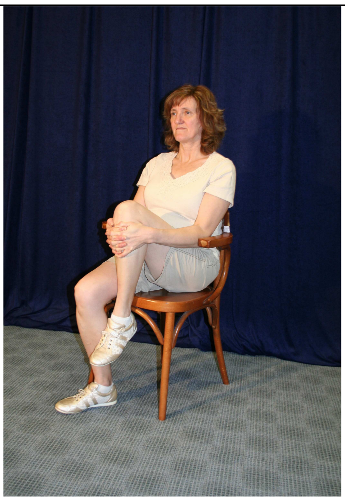	In sitting, bring ………… knee up towards chest<br><br>Hold lower leg just below the knee<br><br>Try and bend your knee further by gently pulling your lower leg towards you<br><br>Hold for __________ seconds<br><br>Repeat ☐ ☐ ☐ times
B	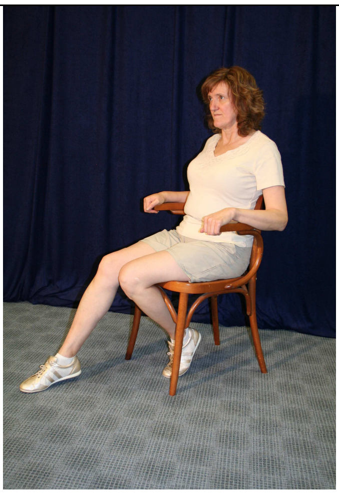	Sit on a chair<br><br>Bend your ……….. knee under the chair as far as you can, keeping the foot on the ground.<br><br>Hold for _________ seconds<br><br>Return to the starting position
C	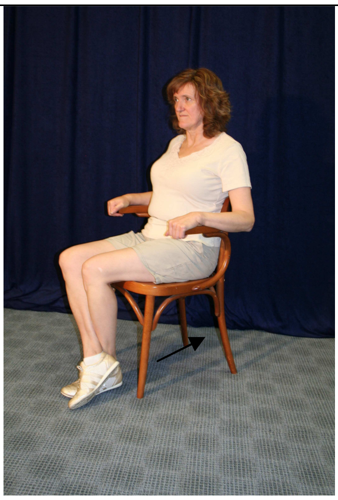	Sit on a chair<br><br>Bend your ……….. knee under the chair as far as you can, keeping the foot on the ground.<br><br>Place your opposite lower leg in front of your shin and push your knee back even further with your unaffected/opposite leg<br><br>Hold for __________ seconds<br>Return to the starting position<br><br>Repeat ☐ ☐ ☐ times

STRETCH EXERCISES

	Picture	Description
D	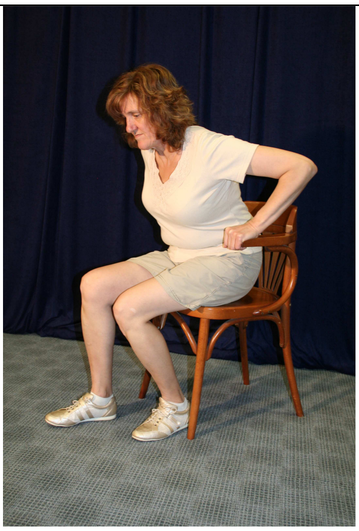	Sitting on a chair<br><br>Bend your .... knee under the chair<br><br>Keeping the ……….. foot on the ground, shuffle forward in the chair to increase the bend in that knee<br><br>Hold for ________ seconds<br><br>Return to the starting position
E	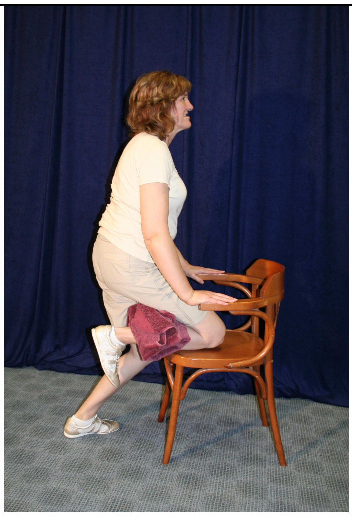	Place your .... knee on a chair.<br>Place a rolled towel behind the knee<br><br>Gently bend your knee by sitting back onto your heel<br><br>Hold this position for _________ seconds<br><br>Return to starting position<br><br>Repeat ☐ ☐ ☐ times
F	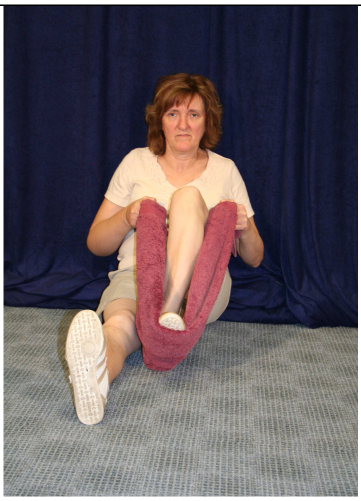	Sit with your back supported against a wall. Bend your .... knee<br><br>Hook a towel around your foot.<br><br>Bend your knee as far as possible<br>Gently pulling on the towel try and bend your knee even further, bringing your heel towards your buttock<br>Hold for __________ seconds<br><br>Repeat ☐ ☐ ☐ times

STRETCH EXERCISES

	Picture	Description
G	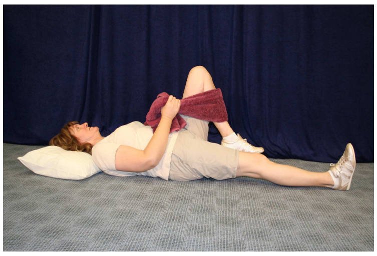	Sit on the floor with your ………… knee bent.<br>Hook a towel around your ………… lower leg<br><br>Lower down onto your back<br><br>Increase the bend in your knee by pulling gently on the band<br><br>Hold this position for _________ seconds<br><br>Repeat times
H	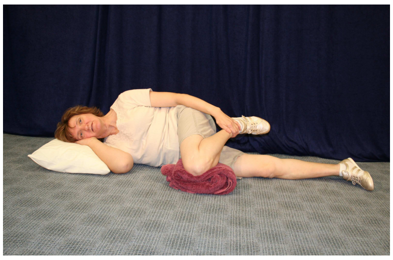	Lie on your ……….. side<br>Bend your uppermost leg and place towel/cushion underneath that knee<br><br>Take hold of ………. ankle<br><br>Increase the bend in your uppermost leg by gently drawing your heel towards your buttock<br><br>Hold this position for _________ seconds<br><br>Repeat times

STRETCH EXERCISES

<table>
<tr><th></th><th colspan="2">Picture and Description</th></tr>
<tr><td>I</td><td colspan="2">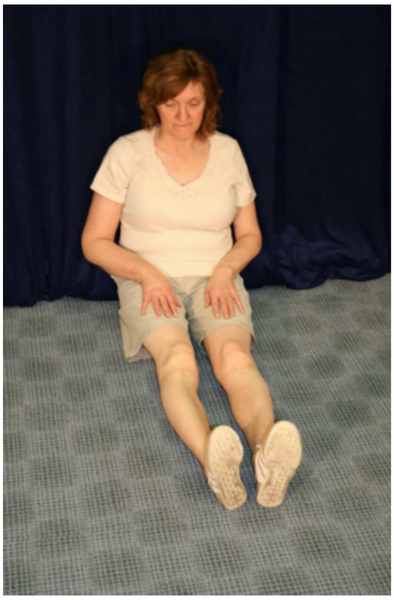 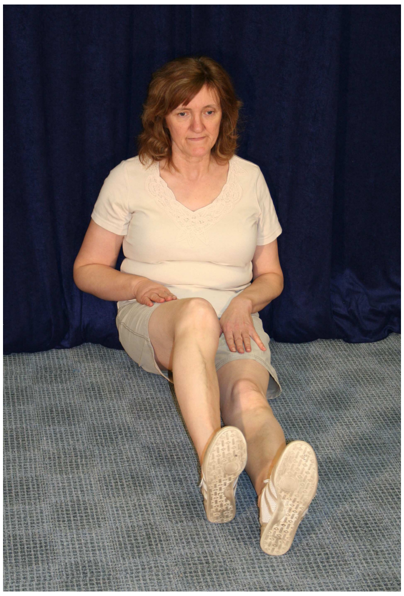 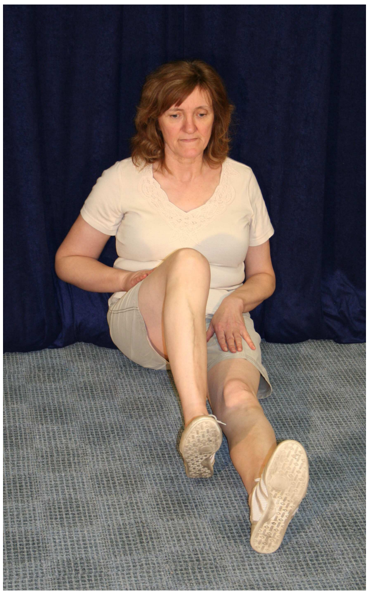

Sit with your back supported against a wall. Slowly bend your .... knee as much as possible, bringing your heel towards your buttock

Hold for ________ seconds

Repeat ☐ ☐ ☐ times</td></tr>
<tr><td>J</td><td>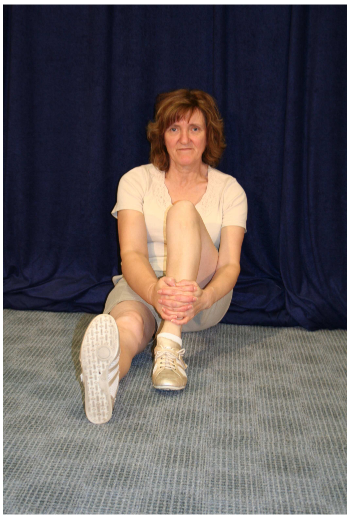</td><td>Sit with your back supported against a wall. Bend your .... knee

Grasp your lower leg around the shin and try and bend your knee as much as possible, bringing your heel towards your buttock

Hold for ________ seconds

Repeat ☐ ☐ ☐ times</td></tr>
</table>

STRETCH EXERCISES

	Picture and Description	
K	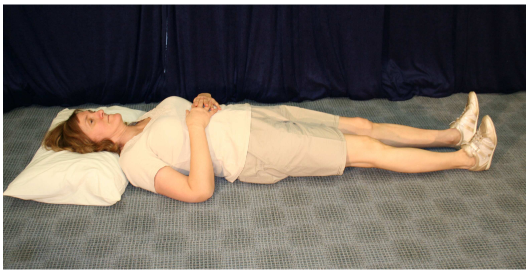<br>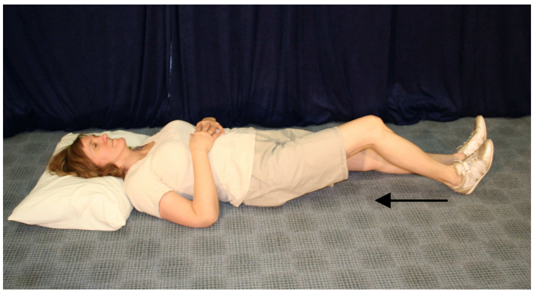<br>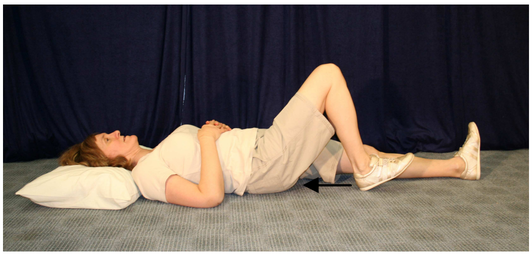	Start in lying position on the floor<br><br>Slowly bend your ……………… knee bringing your heel towards your buttock.<br><br>Keep the heel of the foot resting on the floor as you bend your knee<br><br>Hold your knee in a fully bent position for …………… seconds<br><br>Return your leg to the starting position again<br><br>Repeat ☐ ☐ ☐ times
L	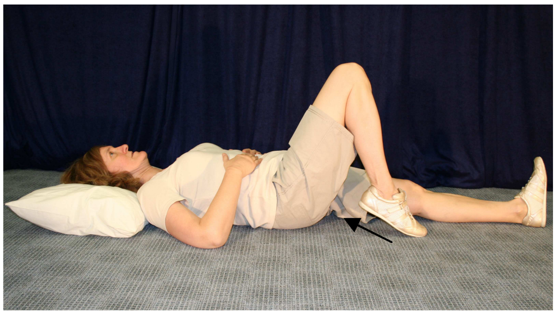<br>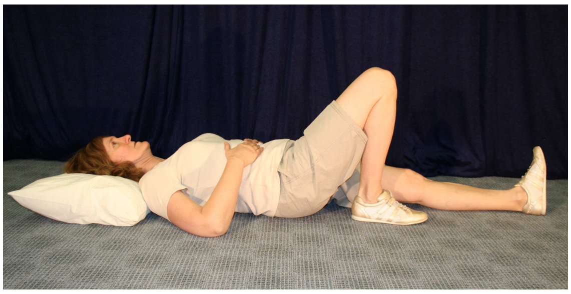	To try and bend your knee even further, lift onto the ball of your foot and continue to slide your foot towards your buttock.<br><br>At the limit of the stretch place your foot flat on the ground again.<br><br>Hold this position of stretch for ………….. seconds<br><br>Repeat ☐ ☐ ☐ times

STRETCH EXERCISES

	Picture and Description	
M	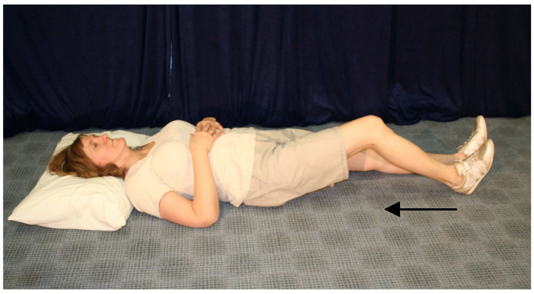<br>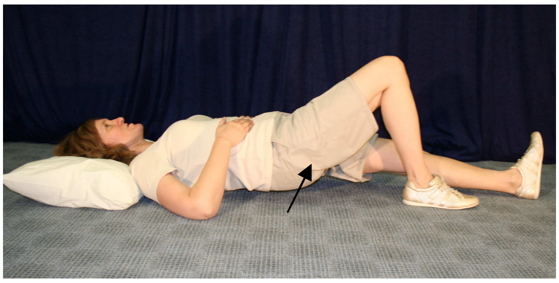<br>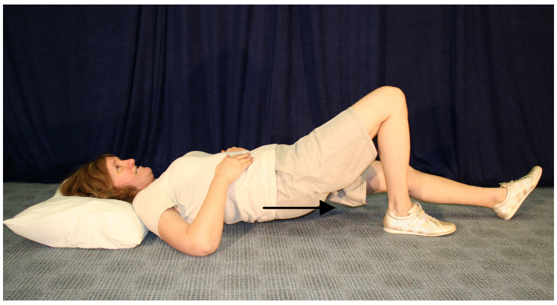<br>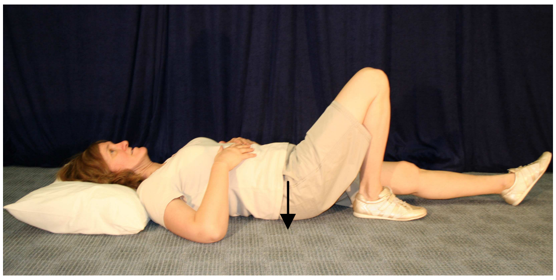	Start in lying position on the floor<br><br>Slowly bend your ……………… knee bringing your heel towards your buttock.<br><br>Keep the heel of the foot resting on the floor as you bend your knee<br><br>To try and bend your knee even further, lift your bottom off the floor and shuffle your bottom and upper body towards your foot.<br><br>Lower your bottom to the ground again<br><br>Hold your knee in a this bent position for …………… seconds<br><br>Straighten out your leg again to return it to the starting position<br><br>Repeat ☐ ☐ ☐ times

STRETCH EXERCISES

	Picture	Description
N	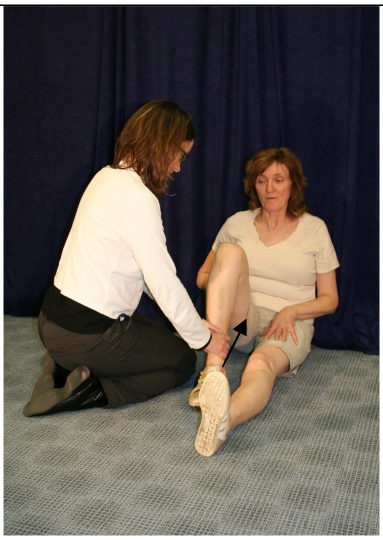	Sit with your back supported against a wall.<br>Slowly bend your .... knee as much as possible, bringing your heel towards your buttock<br><br>A partner can add a further stretch to your knee by holding the lower leg and gently pushing it<br><br>Your partner holds the position of stretch for ………… seconds<br><br>Gently straighten the leg again<br><br>Repeat ☐ ☐ ☐ times
O	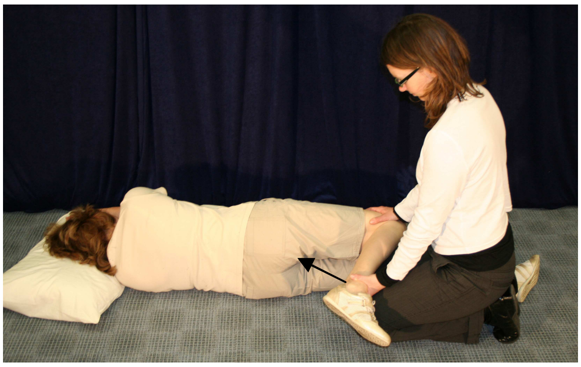	Lie on your .... side<br>Bend your uppermost leg<br><br>A partner can support this leg on their thigh and add a further stretch by gently pushing on your lower leg.<br><br>Hold this position for ________ seconds<br><br>Repeat ☐ ☐ ☐ times

STRETCH EXERCISES

P	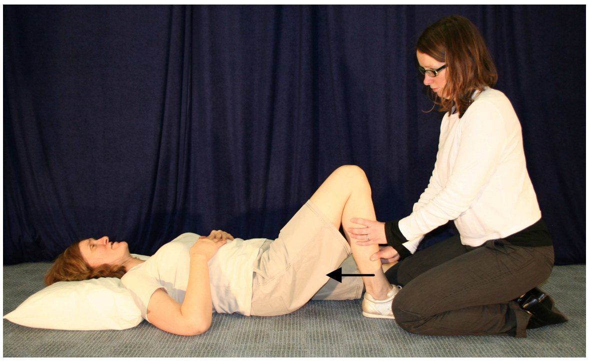	Start in lying position on the floor<br><br>Slowly bend your ………………. knee bringing your heel towards your buttock.<br><br>A partner can add a further stretch to your knee by holding the lower leg and gently pushing it<br><br>Hold this position of stretch for …………. seconds<br><br>Repeat ☐ ☐ ☐ times
