# Supplementary material for: Impairment-targeted exercises for older adults with knee pain: protocol for a proof-of-principle study
Source: BMC Musculoskelet Disord. 2011 Jan 7;12:2. doi: 10.1186/1471-2474-12-2 (PMC3025930; doi:10.1186/1471-2474-12-2)
Supplement: Additional file 2 — "Quadriceps strengthening exercises". (Images of a model performing each of the exercises, together with a verbal description of how to perform each of the exercises). [file 1471-2474-12-2-S2.PDF]

STRENGTH EXERCISES
LEVEL 1

	Picture	Description
1 A	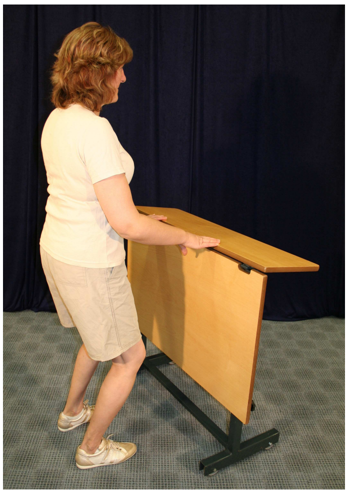	Stand in front of worktop with hands resting on surface for support. Place feet shoulder width apart<br><br>Slowly bend knees until both knee caps are directly in line with first 3 toes.<br><br>Slowly straighten up again<br><br>Repeat ☐ ☐ ☐ times
1 B	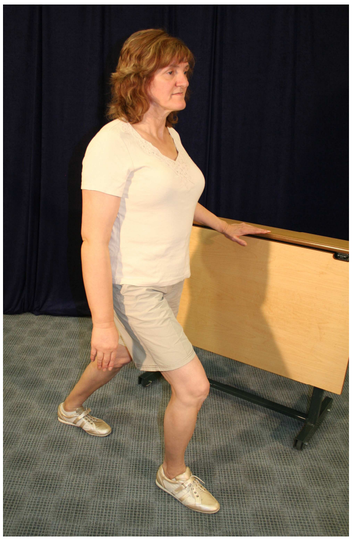	Stand beside a worktop with hand resting on surface for support.<br><br>Step forward with .... foot into a lunge position, keeping the knee cap in line with your toes<br><br>Hold this position for ________ seconds<br><br>Step back so feet are together again<br><br>Repeat ☐ ☐ ☐ times
1 C	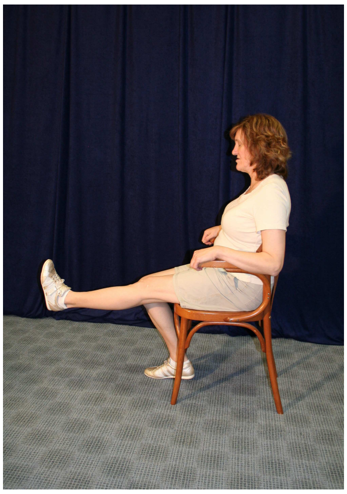	Sit up straight in a chair<br><br>Slowly straighten …………… leg<br><br>Toes should point towards the ceiling<br><br>Hold this position for ________ seconds<br><br>Lower your leg again slowly.<br><br>Repeat ☐ ☐ ☐ times

STRENGTH EXERCISES
LEVEL 1

1 D

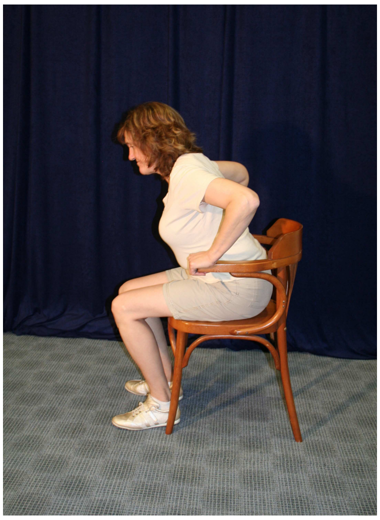 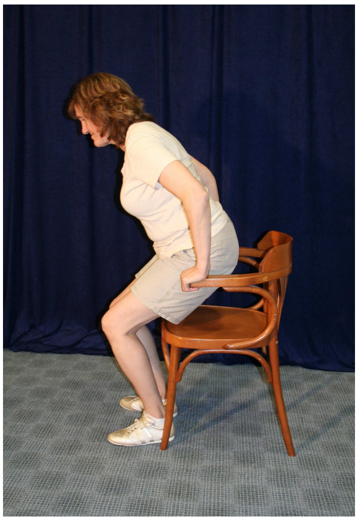 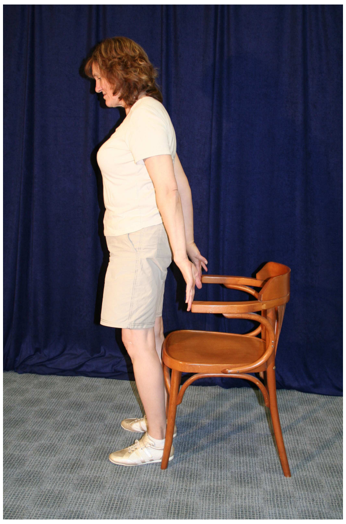

Sit in a chair.

Stand up slowly, using your hands to assist you.

Lower yourself down to the chair again slowly

Repeat ☐ ☐ ☐ times

STRENGTH EXERCISES
LEVEL 2

	Picture	Description
2 A	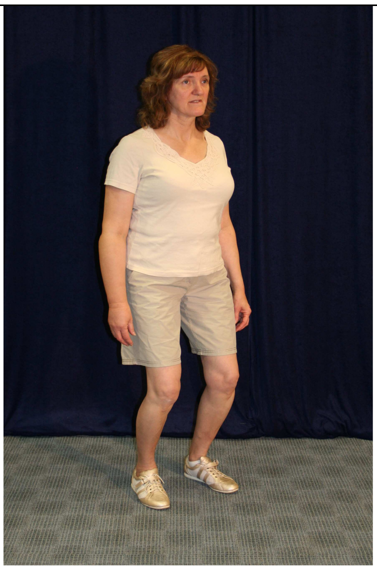	Place feet shoulder width apart<br><br>Look straight ahead<br><br>Slowly bend knees until both knee caps are directly in line with first three toes.<br><br>Slowly straighten up again<br><br>Repeat ☐ ☐ ☐ times
2 B	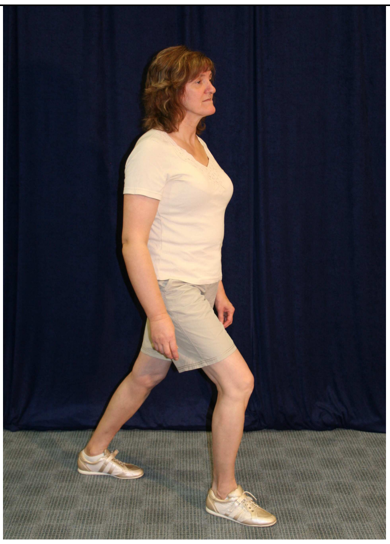	Step forward with .... foot into a lunge position, keeping the knee cap in line with your toes<br><br>Hold this position for ________ seconds<br><br>Step back so feet are together again<br><br>Repeat ☐ ☐ ☐ times
2 C	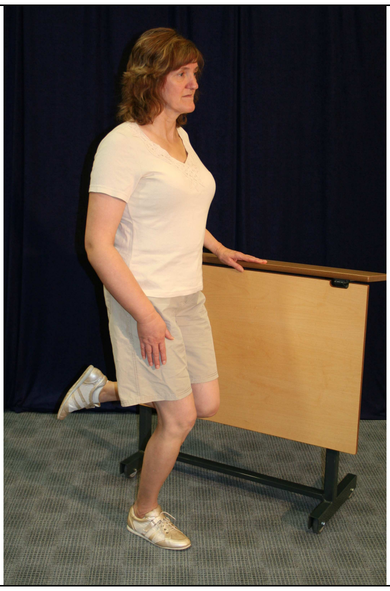	Stand beside worktop with hand lightly resting on surface for support<br>Look straight ahead<br><br>Stand on .... leg<br>Slowly bend knee until knee cap is directly over toes.<br><br>Slowly straighten up again<br><br>Repeat ☐ ☐ ☐ times

STRENGTH EXERCISES
LEVEL 2

	Picture	Description
2 D	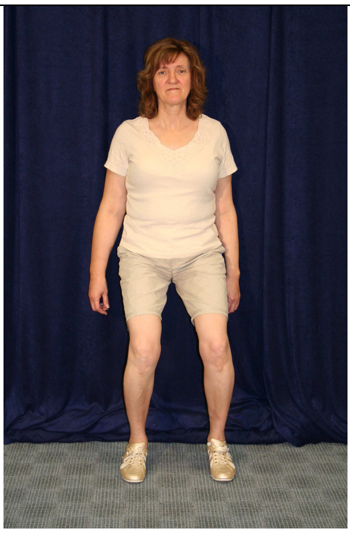	Stand leaning with your back against the wall and your feet approximately 12 inches from the wall<br><br>Keeping your feet in line with your hips, slowly slide down the wall into a half sitting position until your knee caps are directly over your toes<br><br>Hold this position for ___________ seconds, then return to standing again
2 E	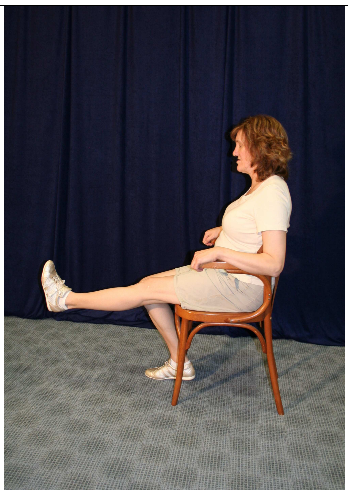	Sit up straight in a chair<br>Slowly straighten your ……….. leg<br>Toes should point towards the ceiling<br><br>Hold this position for __________ seconds<br><br>Lower your leg again slowly<br><br>Repeat times
2 F	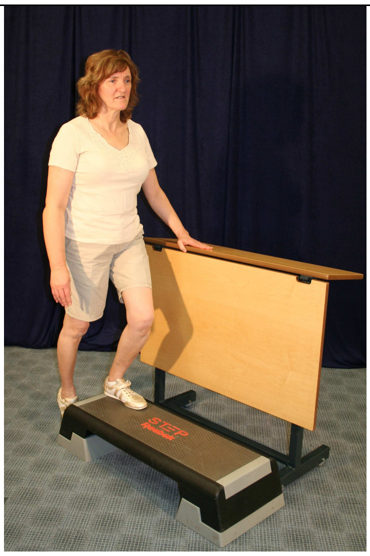	Stand at the bottom of the stairs holding onto the stair rail or wall for support<br><br>Step up onto the bottom step with your ……….. leg<br><br>Step down slowly with the opposite leg.<br>Look straight ahead as you step up and down<br><br>Repeat times

<table>
<tr>
<td>2 G</td>
<td>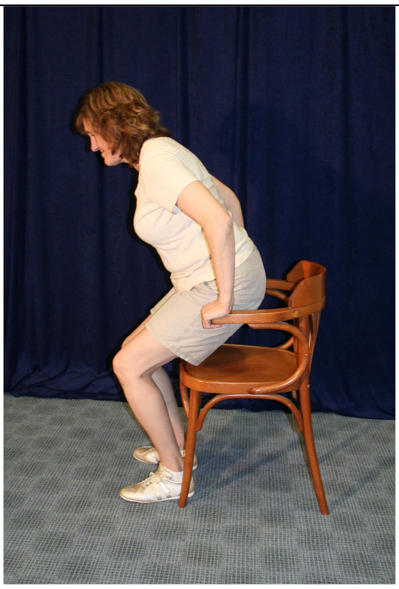</td>
<td>Sit in a chair. Stand up slowly, using one hand to assist you.

Repeat ☐ ☐ ☐ times</td>
</tr>
<tr>
<td>2 H</td>
<td colspan="2">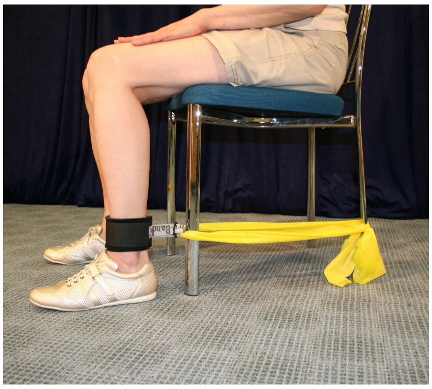 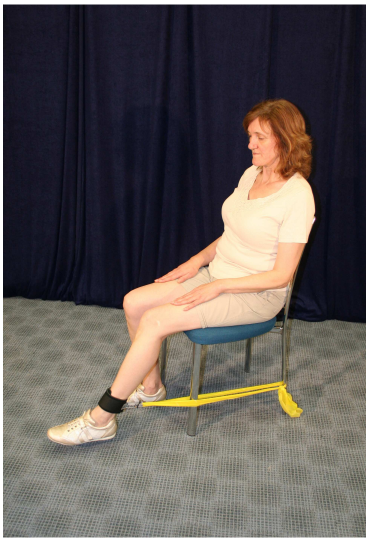 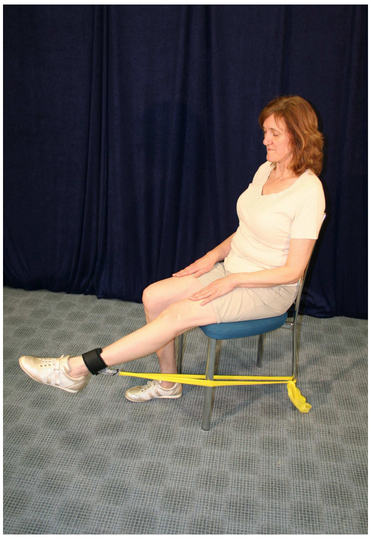

Sit in a chair, with the ………….TheraBand securely tied to the back leg of the chair as shown above

Attach the velcro ankle cuff around your ankle, with the buckle at the back of the lower leg (see first picture above)

Keeping your thigh flat on the chair, slowly straighten your knee. Hold your knee straight for _________ seconds.

Slowly return to the starting position

Repeat ☐ ☐ ☐ times

Release your leg from the theraband ankle cuff before standing up again</td>
</tr>
</table>

STRENGTH EXERCISES
LEVEL 3

	Picture	Description
3 A	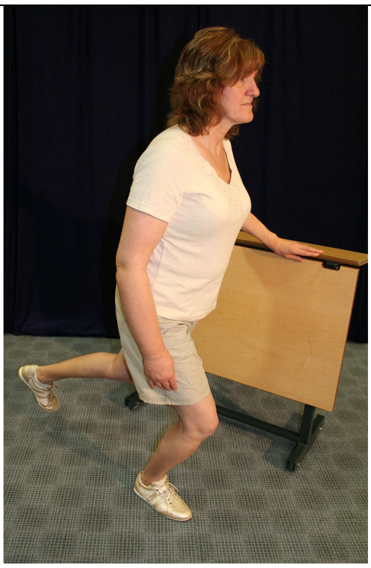	Stand beside worktop with hand lightly resting on surface for support<br><br>Step forward with .... foot into a lunge position, keeping the knee cap in line with your toes<br><br>Hold this position for _________ seconds<br><br>Step back so feet are together again<br><br>Repeat ☐ ☐ ☐ times
3 B	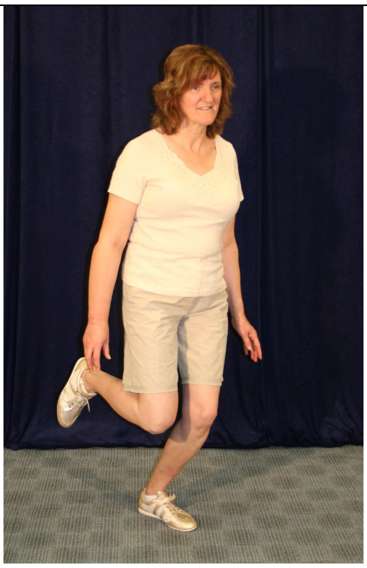	Stand on .... leg<br><br>Look straight ahead<br><br>Slowly bend knee until kneecap is in line with your toes.<br><br>Slowly straighten up again<br><br>Repeat ☐ ☐ ☐ times
3 C	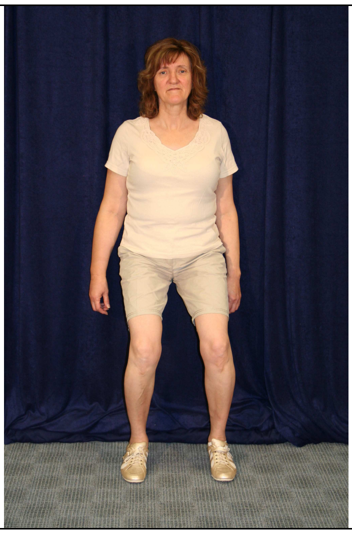	Stand leaning with your back against the wall and your feet approximately 12 inches from the wall<br><br>Keeping your feet in line with your hips, slowly slide down the wall into a half sitting position until your knee caps are in line with your toes<br><br>Hold this position for ____________ seconds, then return to standing again

STRENGTH EXERCISES
LEVEL 3

	Picture	Description
3 D	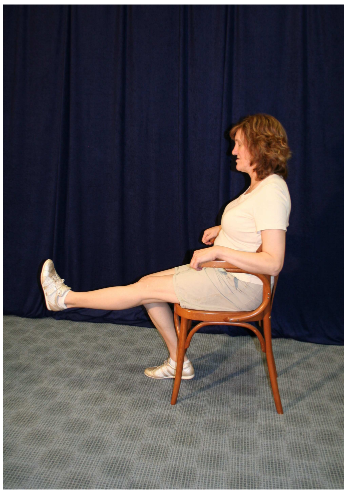	Sit up straight in a chair<br><br>Slowly straighten your ……….. leg<br><br>Toes should point towards the ceiling<br><br>Hold this position for _________ seconds<br><br>Lower your leg again slowly
3 E	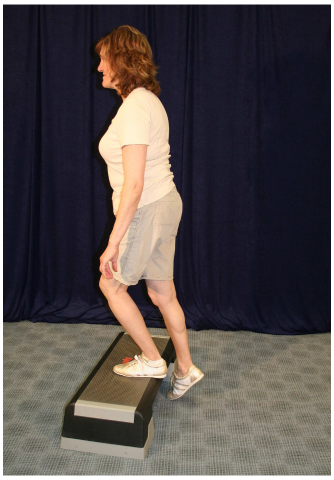 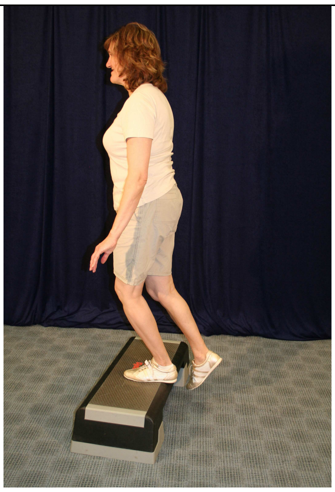 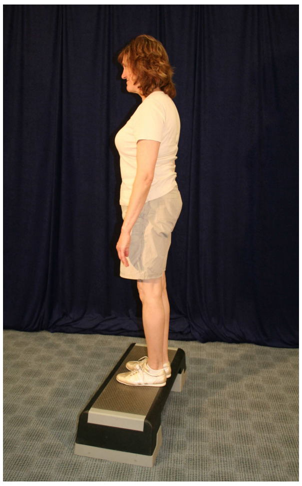<br><br>Stand at the bottom of the stairs. Step up onto the bottom step with your ……….. leg, without holding onto the support<br><br>Step down slowly with the opposite leg.<br>Look straight ahead as you step up and down<br><br>Repeat ☐ ☐ ☐ times	

STRENGTH EXERCISES
LEVEL 3

	Picture and Description
3 F	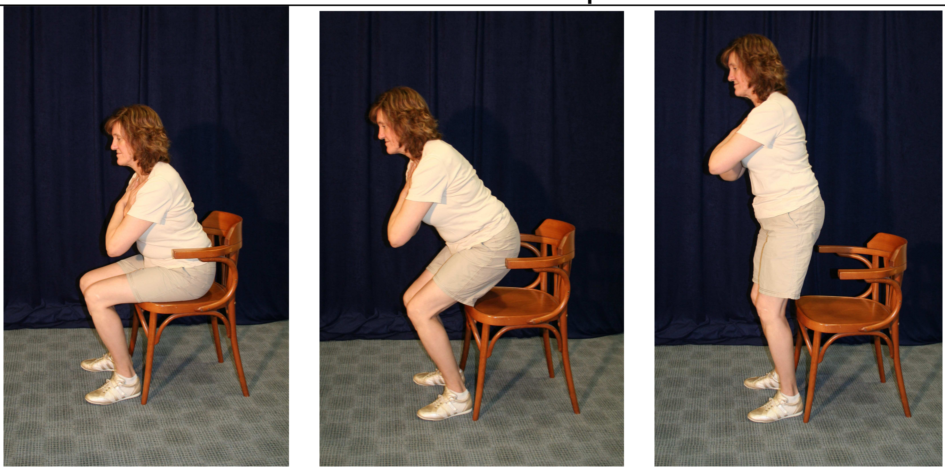<br>Sit in a chair. Stand up slowly, without using your hands to assist you.<br><br>Lower yourself down to chair again slowly<br><br>Repeat ☐ ☐ ☐ times
3 G	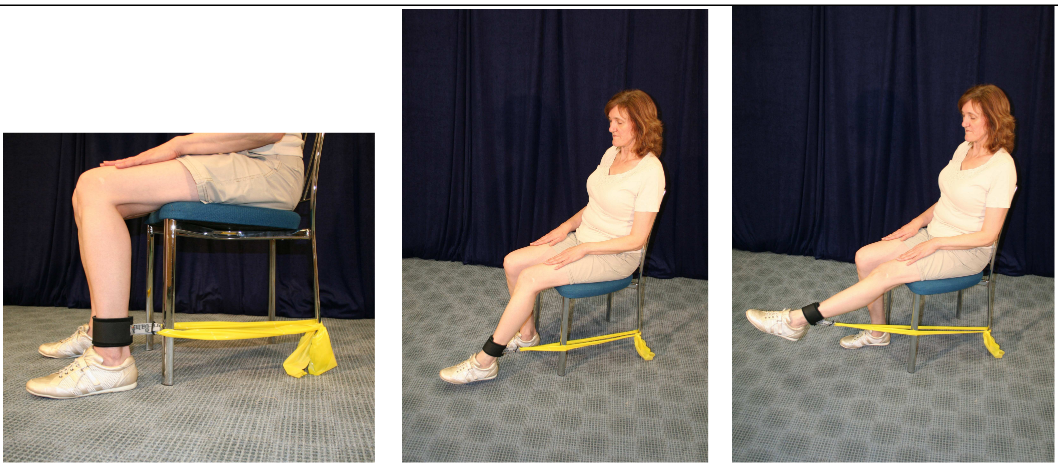<br>Sit in a chair, with the …………TheraBand securely tied to the back leg of the chair as shown above<br><br>Attach the velcro ankle cuff around your ankle, with the buckle at the back of the lower leg (see first picture above)<br><br>Keeping your thigh flat on the chair, slowly straighten your knee. Hold your knee straight for _________ seconds. Slowly return to the starting position<br><br>Repeat ☐ ☐ ☐ times<br><br>Release your leg from the theraband ankle cuff before standing up again

STRENGTH EXERCISES
LEVEL 4

	Picture	Description
4 A	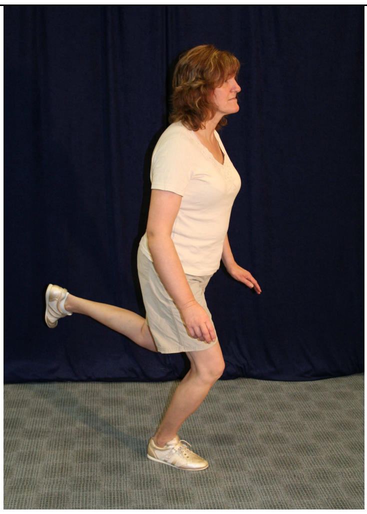	Step forward with .... foot into a lunge position, keeping the knee cap in line with your toes<br><br>Hold this position for ________ seconds<br><br>Step back so feet are together again<br><br>Repeat ☐ ☐ ☐ times
4 B	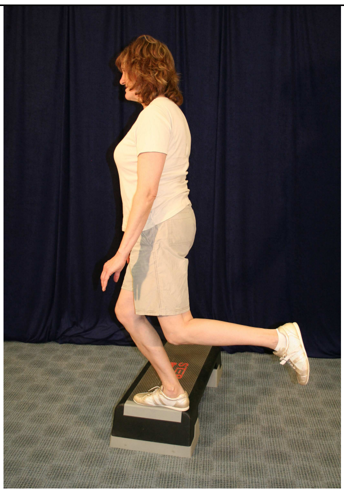	Stand with ………… leg on a step/stairs<br><br>Bend this knee so that the other leg lowers to the floor - do not let it touch the floor<br><br>Keep your body weight directly over the leg you are standing on and look straight ahead<br><br>Slowly straighten up again<br><br>Repeat ☐ ☐ ☐ times
4 C	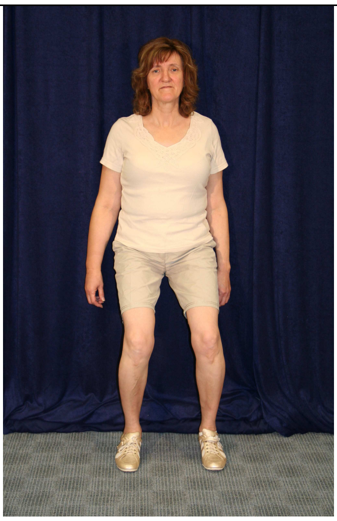	Stand leaning with your back against the wall and your feet approximately 12 inches from the wall<br><br>Keeping your feet in line with your hips, slowly slide down the wall into a half sitting position until your knee caps are in line with your toes<br><br>Hold this position for ___________ seconds, then return to standing again

STRENGTH EXERCISES
LEVEL 4

	Picture	Description
4 D	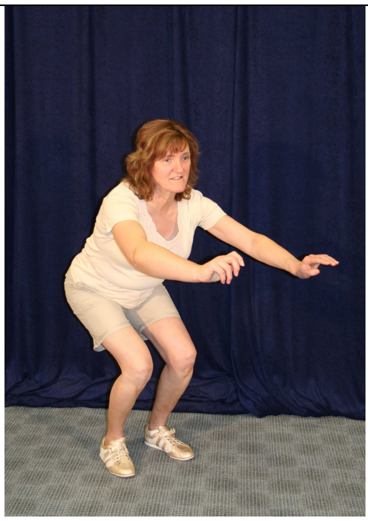	Stand with feet hip width apart.<br><br>Slowly squat down bending knees to a right angle. Keep your weight over your heels and your back straight and do not let your knees come closer together.<br><br>Hold this position for __________ seconds, then return to standing again<br><br>Repeat ☐ ☐ ☐ times
4 E	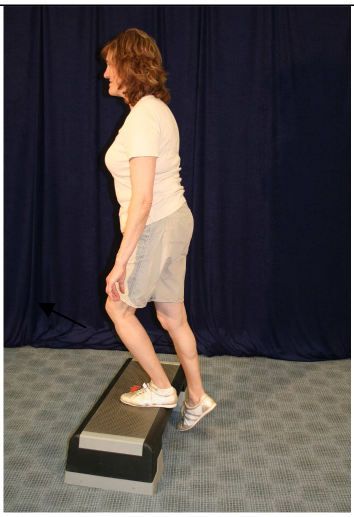	Stand at the bottom of the stairs. Step up onto the bottom step with your ……….. leg, without holding onto the stair-rail.<br><br>Step down slowly with the opposite leg.<br><br>Look straight ahead as you step up and down<br><br>Repeat ☐ ☐ ☐ times

STRENGTH EXERCISES – LEVEL 4

	Picture and Description
4 F	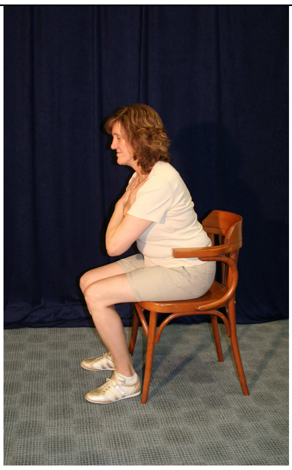 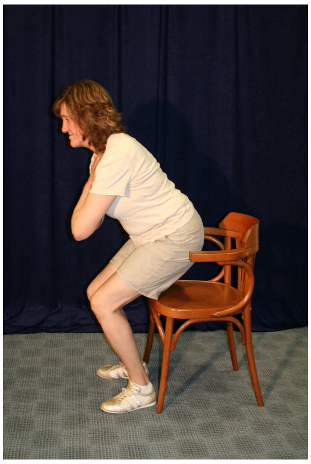 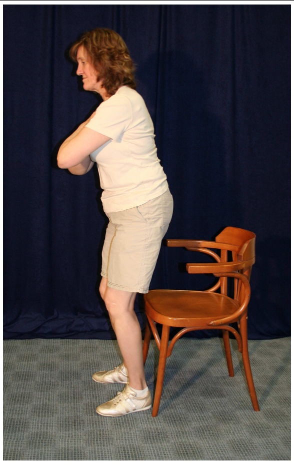<br>Sit in a chair. Stand up slowly, without using your hands to assist you.<br><br>Lower yourself down to chair again slowly<br><br>Repeat ☐ ☐ ☐ times
4 G	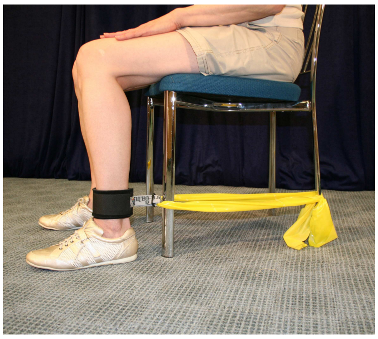 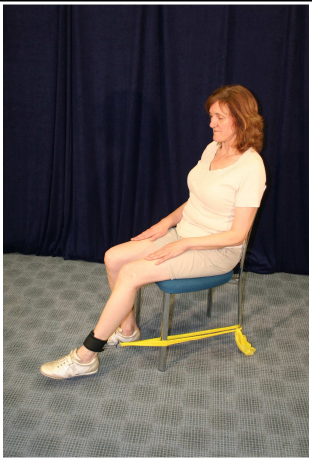 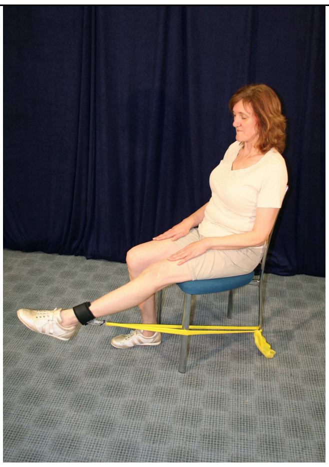<br>Sit in a chair, with the …………TheraBand securely tied to the back leg of the chair as shown above<br><br>Attach the velcro ankle cuff around your ankle, with the buckle at the back of the lower leg (see first picture above)<br><br>Keeping your thigh flat on the chair, slowly straighten your knee. Hold your knee straight for _________ seconds. Slowly return to the starting position<br><br>Repeat ☐ ☐ ☐ times<br><br>Release your leg from the theraband ankle cuff before standing up again
