# Supplementary material for: Impairment-targeted exercises for older adults with knee pain: protocol for a proof-of-principle study
Source: BMC Musculoskelet Disord. 2011 Jan 7;12:2. doi: 10.1186/1471-2474-12-2 (PMC3025930; doi:10.1186/1471-2474-12-2)
Supplement: Additional file 3 — "Balance retraining exercises". (Images of a model performing each of the exercises, together with a verbal description of how to perform each of the exercises). [file 1471-2474-12-2-S3.PDF]

BALANCE EXERCISES
LEVEL 1

	Picture	Description
1 A	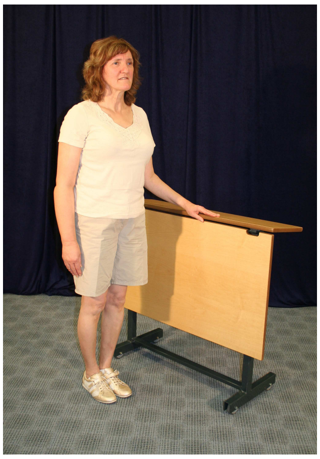	Stand beside a worktop. Rest hand on surface for support if needed. Stand with both feet together<br><br>Look straight ahead<br><br>Hold this position for _________ seconds<br><br>Repeat ☐ ☐ ☐ times<br><br>Progress to not holding onto the worktop.
1 B	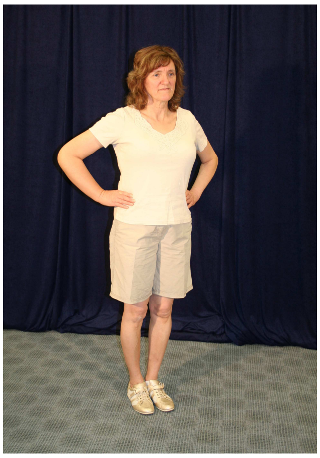	Stand with your feet together.<br><br>Place your hands on your hips<br><br>Hold this position for ________ seconds<br><br>Make a note of how long you can maintain your balance<br><br>Repeat ☐ ☐ ☐ times
1 C	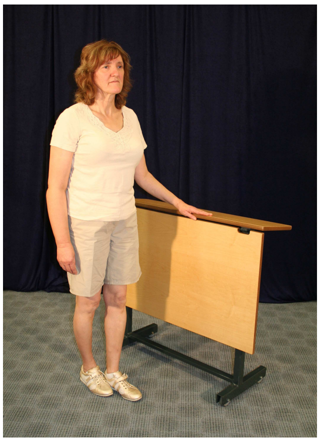	Stand beside a worktop with hand lightly resting on surface for support.<br><br>Place feet in “semi-tandem” position so the toes of one foot are level with the inside arch of the other foot.<br><br>Hold this position for _________ seconds<br><br>Repeat ☐ ☐ ☐ times

BALANCE EXERCISES
LEVEL 1

	Picture	Description
1 D	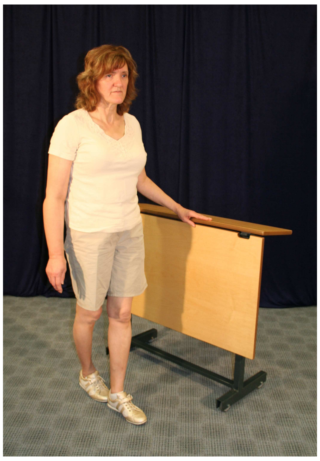	Stand beside a worktop with hand/s lightly resting on surface for support.<br><br>Place feet in “tandem” position so the toes of one foot are touching the heel of the other foot.<br><br>Hold this position for up to 30 seconds<br><br>Repeat ☐ ☐ ☐ times

BALANCE EXERCISES
LEVEL 2

	Picture	Description
2 A	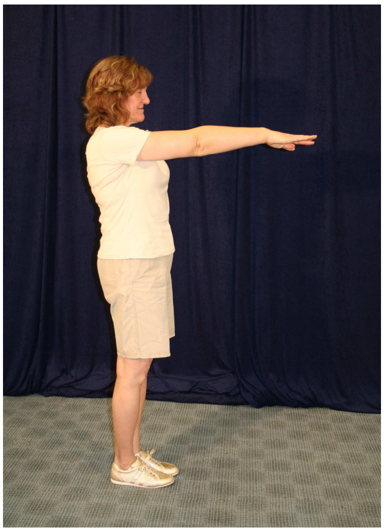	Stand with your feet together<br><br>Raise arms in front so that your hands are level with your shoulders<br><br>Hold this position for __________ seconds<br><br>Make a note of how long you can maintain your balance<br><br>Repeat ☐ ☐ ☐ times
2 B	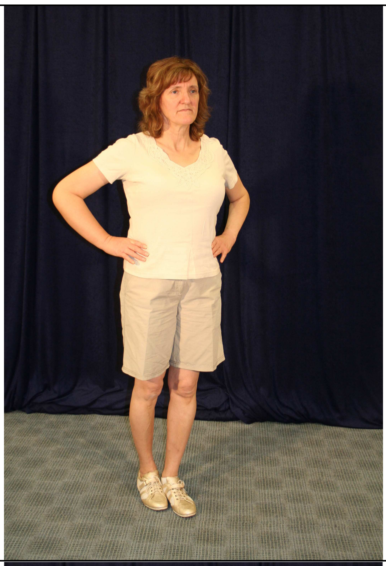	Stand with feet in semi-tandem position: heel of one foot should rest beside arch of other foot. Place your hands on your hips<br><br>Look straight ahead<br><br>Hold this position for _________ seconds<br><br>Record how long you are able to maintain your balance in this position<br><br>Repeat ☐ ☐ ☐ times
2 C	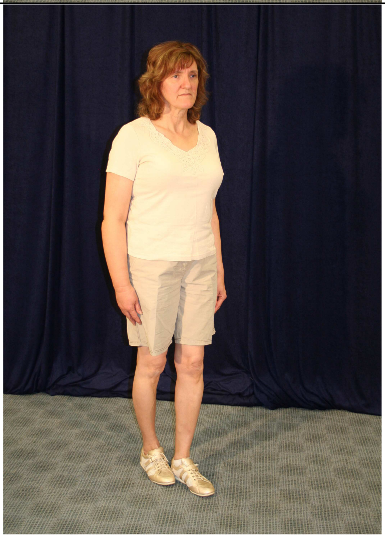	Stand with feet in semi-tandem position: heel of one foot should rest beside arch of other foot<br><br>Look straight ahead<br><br>Hold this position for _________ seconds<br><br>Record how long you are able to maintain your balance in this position<br><br>Repeat ☐ ☐ ☐ times

BALANCE EXERCISES
LEVEL 2

	Picture	Description
2 D	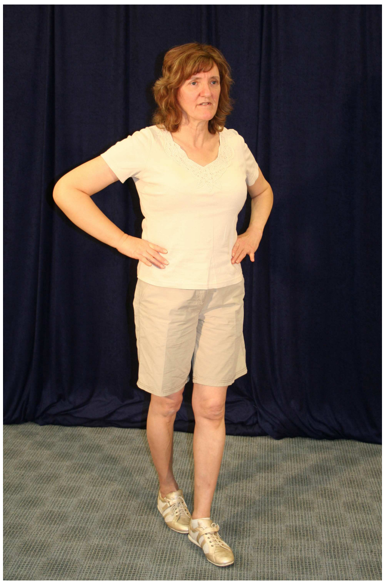	Stand in “tandem” position with your toes of one foot touching heel of other foot<br><br>Place your hands on your hips<br><br>Hold this position for __________ seconds<br><br>Make a note of how long you can maintain your balance<br><br>Repeat ☐ ☐ ☐ times
2 E	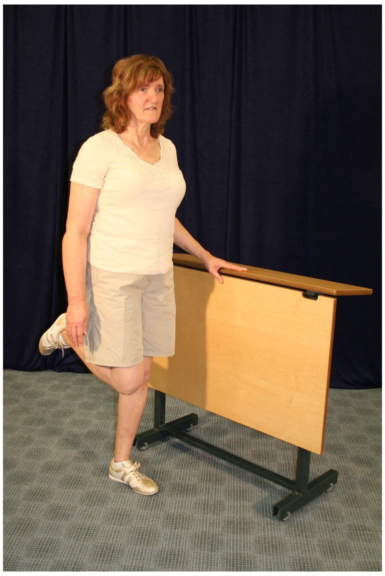	Stand facing worktop with hand/s lightly resting on surface for support<br><br>Stand on one leg<br><br>Look straight ahead<br><br>Hold this position for _________ seconds<br><br>Record how long you are able to maintain your balance in this position<br><br>Repeat ☐ ☐ ☐ times

BALANCE EXERCISES
LEVEL 3

	Picture	Description
3 A	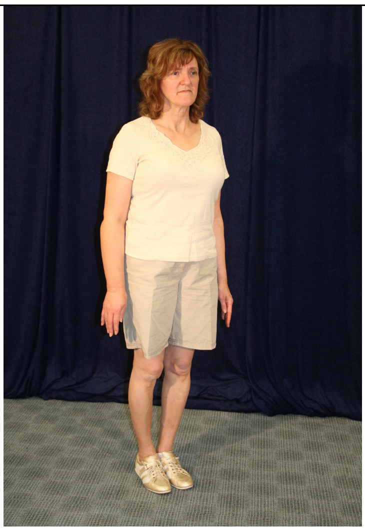	Stand with feet together.<br><br>Close one eye<br><br>Hold this position for ________ seconds<br><br>Make a note of how long you can maintain your balance
3 B	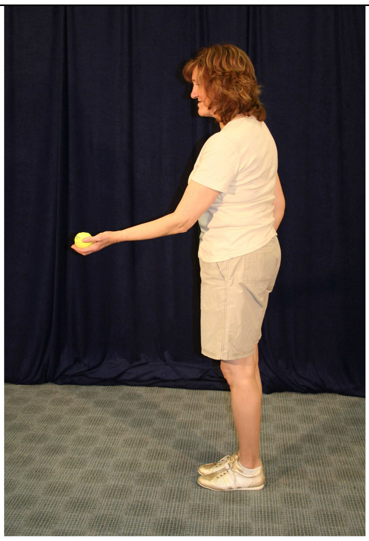	Stand with feet together.<br><br>Throw a small ball against the wall<br><br>Repeat ☐ ☐ ☐ times
3 C	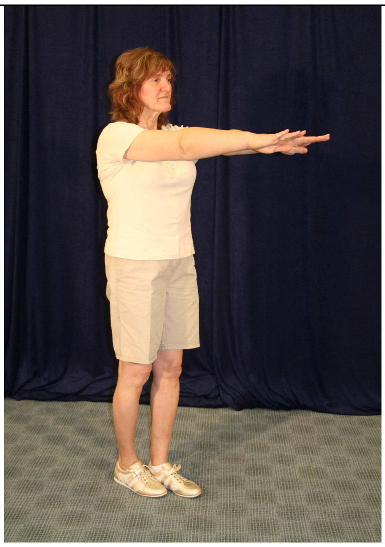	Stand with feet in semi-tandem position: heel of one foot should rest beside arch of other foot<br>Look straight ahead<br><br>Raise arms in front so that hands are level with your shoulders<br><br>Hold this position for _________ seconds<br><br>Record how long you are able to maintain your balance in this position

BALANCE EXERCISES
LEVEL 3

	Picture	Description
3 D	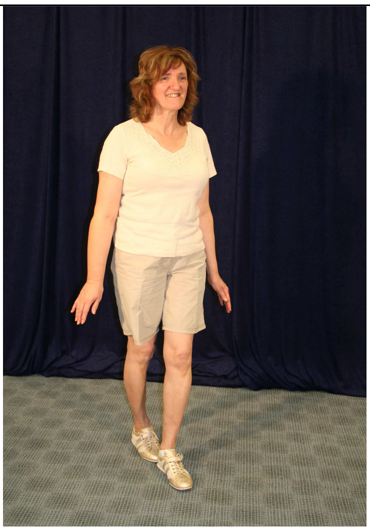	Stand in “tandem” position with your toes of one foot touching heel of other foot<br><br>Without moving your feet, slowly transfer your weight onto the front foot, then onto the back foot.<br><br>Repeat ☐ ☐ ☐ times
3 E	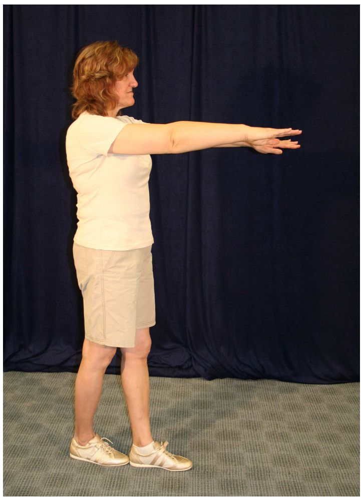	Stand in “tandem” position with your toes of one foot touching heel of other foot.<br><br>Raise your arms in front so that hands are level with your shoulders<br>Look straight ahead<br><br>Hold this position for __________ seconds<br><br>Make a note of how long you can maintain your balance
3 F	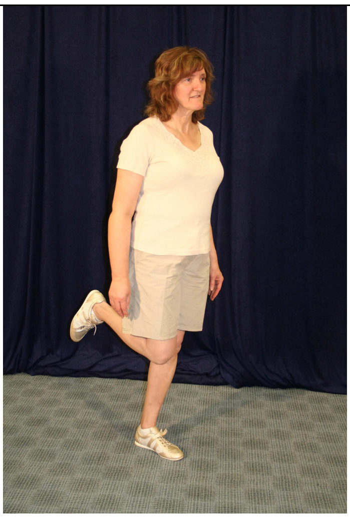	Stand on ………… leg<br><br>Look straight ahead<br><br>Hold this position for __________ seconds<br><br>Record how long you are able to maintain your balance in this position<br><br>Repeat with hands on your hips<br>Hold for __________ seconds

BALANCE EXERCISES
LEVEL 3

	Picture	Description
3 G	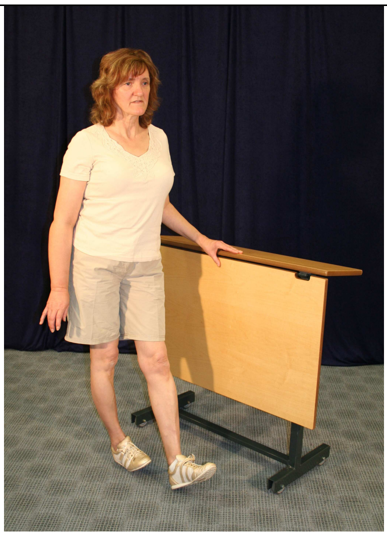	Stand beside worktop with hands lightly resting on surface for support<br><br>Look straight ahead<br><br>Transfer your weight back onto your heels, raising front of both feet off the ground<br><br>Walk forward 10 steps on your heels<br><br>Repeat ☐ ☐ ☐ times
3 H	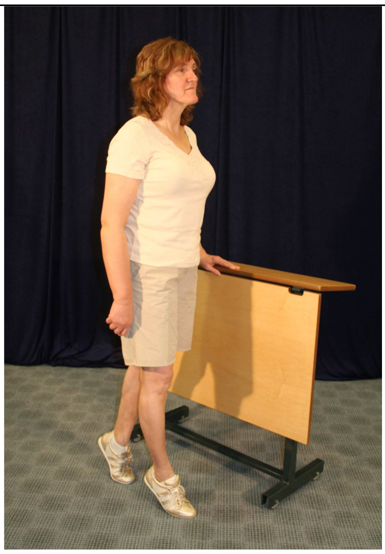	Stand beside worktop with hands lightly resting on surface for support<br><br>Look straight ahead<br><br>Take weight of both feet onto your toes<br><br>Walk forward 10 steps<br><br>Repeat ☐ ☐ ☐ times
3 I	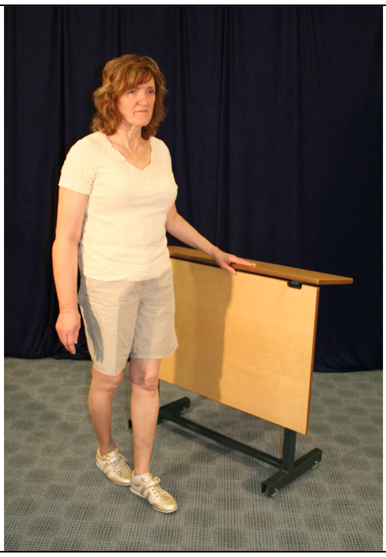	Stand beside a worktop with your hands lightly resting on surface for support<br><br>Look straight ahead<br><br>With feet in "tandem" position walk forward 10 steps then backwards 10 steps<br><br>Repeat ☐ ☐ ☐ times

BALANCE EXERCISES
LEVEL 4

	Picture	Description
4 A	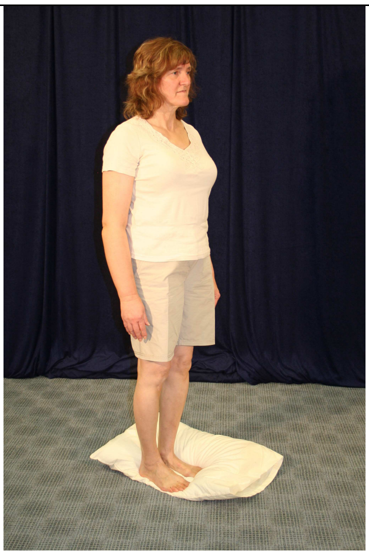	Stand on a cushion/pillow and place feet together.<br><br>Close one eye<br><br>Hold this position for __________ 30 seconds<br><br>Make a note of how long you can maintain your balance
4 B	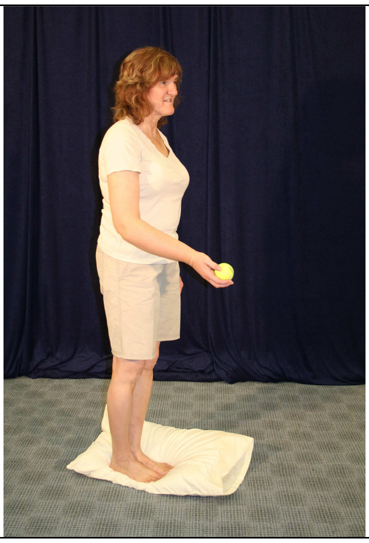	Stand on a cushion/pillow and place feet together.<br><br>Throw a **small ball** against the wall<br><br>Repeat ☐ ☐ ☐ times
4 C	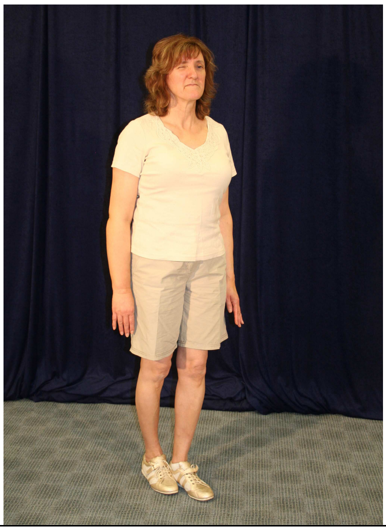	Stand with feet in semi-tandem position: heel of one foot should rest beside arch of other foot<br><br>Look straight ahead<br><br>Close one eye<br><br>Hold this position for _________ seconds<br><br>Record how long you are able to maintain your balance in this position

BALANCE EXERCISES
LEVEL 4

	Picture	Description
4 D	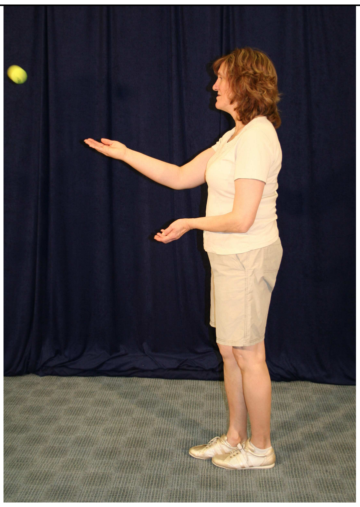	Stand with feet in semi-tandem position: heel of one foot should rest beside arch of other foot<br><br>Throw a small ball against the wall<br><br>Repeat ☐ ☐ ☐ times
4 E	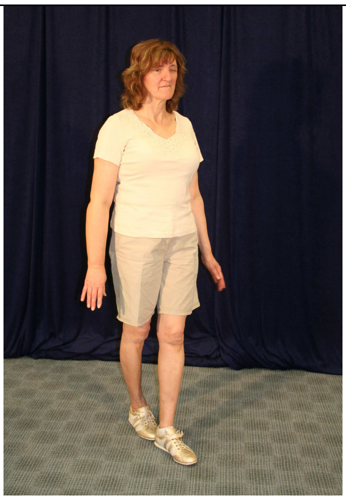	Stand in "tandem" position with your toes of one foot touching heel of other foot<br><br>Close one eye<br><br>Hold this position for __________ seconds<br><br>Record how long you are able to maintain your balance in this position
4 F	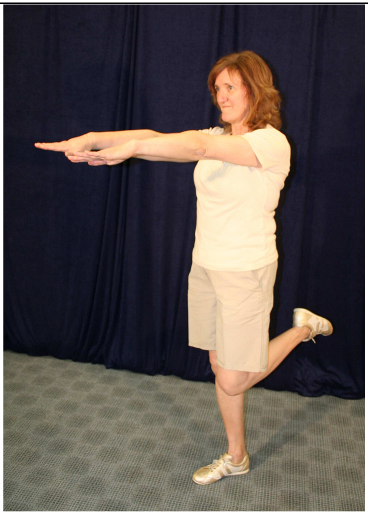	Stand on one leg<br><br>Look straight ahead<br><br>Raise both arms so that hands are level with shoulders<br><br>Hold this position for _________ seconds

BALANCE EXERCISES
LEVEL 4

	Picture	Description
4 G	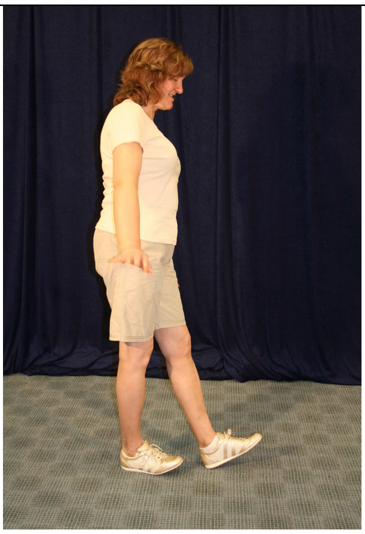	Transfer your weight back onto your heels, raising front of both feet off the ground<br><br>Walk forward 10 steps on your heels<br><br>Repeat ☐ ☐ ☐ times
4 H	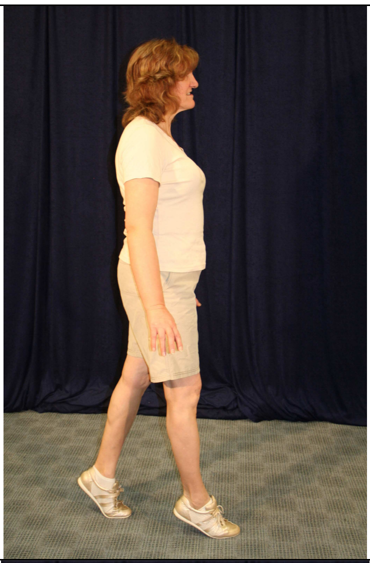	Look straight ahead<br><br>Take weight of both feet onto your toes<br><br>Walk forward 10 steps<br><br>Repeat ☐ ☐ ☐ times
4 i	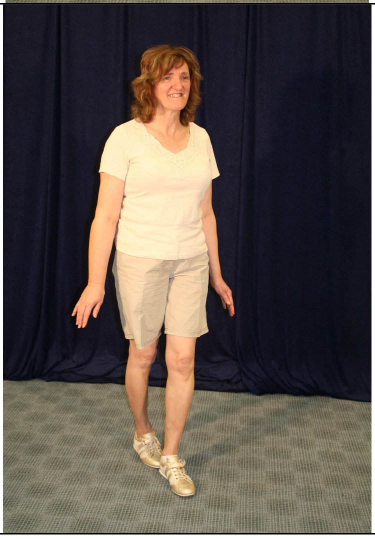	Stand with feet in “tandem” position<br><br>Looking straight ahead, walk forward 10 steps then backwards 10 steps

BALANCE EXERCISES
LEVEL 5

	Picture	Description
5 A	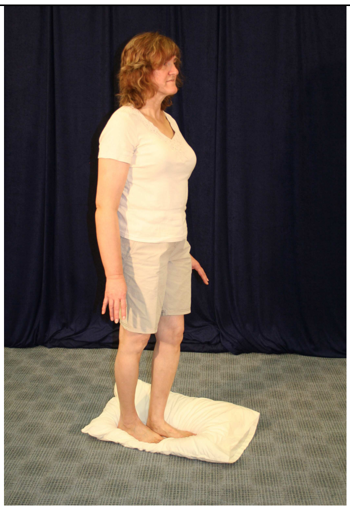	Stand on a cushion/pillow<br>Place feet in "semi tandem" position with the heel of one foot beside the arch of the other foot<br><br>Close one eye<br><br>Hold this position for__________ seconds<br><br>Make a note of how long you are able to maintain your balance in this position
5 B	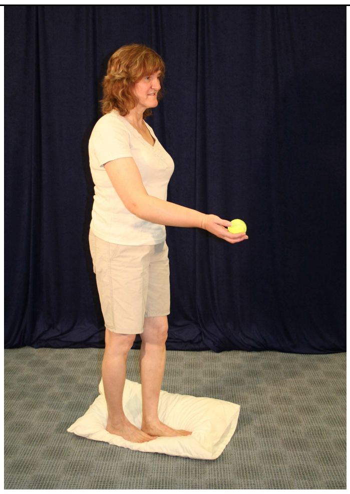	Stand on a cushion/pillow<br><br>Place feet in "semi tandem" position with the heel of one foot beside the arch of the other foot<br><br>Throw a **small ball** against the wall<br><br>Repeat ☐ ☐ ☐ times
5 C	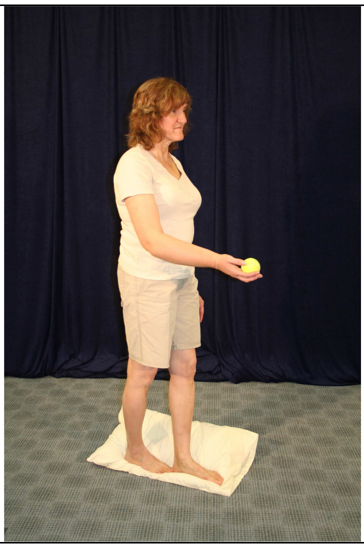	Stand on a cushion/pillow<br><br>Place feet in "tandem" position with your toes of one foot touching heel of other foot<br><br>Throw a **small ball** against the wall<br><br>Repeat ☐ ☐ ☐ times

BALANCE EXERCISES
LEVEL 5

	Picture	Description
5 D	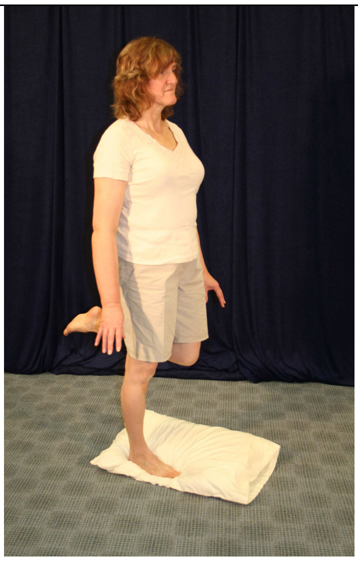	Stand on one leg on a cushion/pillow<br><br>Look straight ahead<br><br>Close one eye<br><br>Hold this position for _________ seconds<br><br>Record how long you are able to maintain your balance in this position
5 E	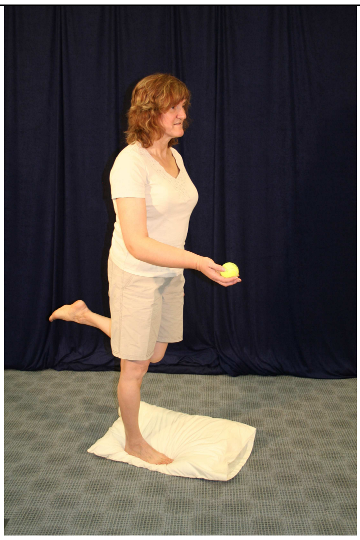	Stand on one leg on a cushion/pillow<br><br>Throw a **small ball** against the wall<br><br>Repeat ☐ ☐ ☐ times
5 F	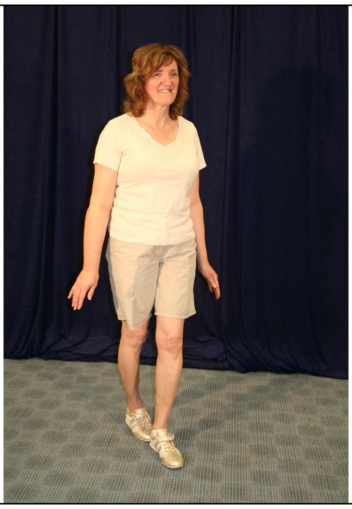	Stand with feet in “tandem” position<br><br>Looking straight ahead, walk forward 20 steps then backwards 20 steps<br><br>Repeat ☐ ☐ ☐ times
