# Supplementary figures and images for: Impairment-targeted exercises for older adults with knee pain: protocol for a proof-of-principle study
Source: BMC Musculoskelet Disord. 2011 Jan 7;12:2. doi: 10.1186/1471-2474-12-2 (PMC3025930; doi:10.1186/1471-2474-12-2)

Chatillon DFX-200 electronic dynamometer with bespoke wall-stabilisation rig

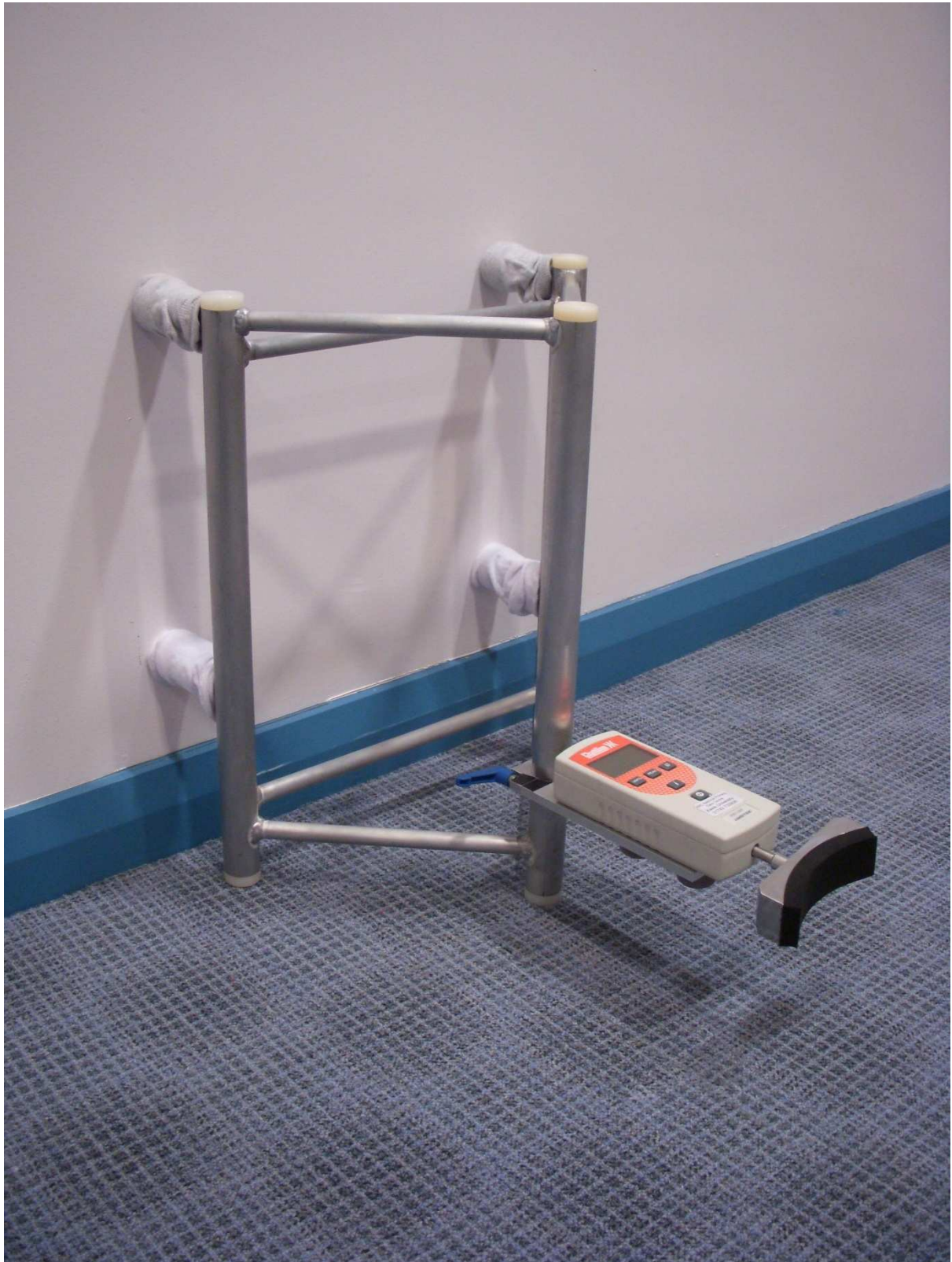

Supplement: Additional file 4 — "Set-up for measurement of isometric quadriceps strengths". (Picture of the Chatillon DFX-200 electronic dynamometer with bespoke wall-stabilisation rig). [file 1471-2474-12-2-S4.PDF]
